# Meeting report: suggestions for studies on future health risks following the Fukushima accident

**DOI:** 10.1186/s12940-015-0013-z

**Published:** 2015-03-19

**Authors:** Tomoko Inamasu, Sara J Schonfeld, Masafumi Abe, Pernille E Bidstrup, Isabelle Deltour, Takashi Ishida, Tetsuo Ishikawa, Ausrele Kesminiene, Tetsuya Ohira, Hitoshi Ohto, Shinichi Suzuki, Isabelle Thierry-Chef, Hirooki Yabe, Seiji Yasumura, Joachim Schüz, Shunichi Yamashita

**Affiliations:** Section of Environment and Radiation, International Agency for Research on Cancer, 150 cours Albert Thomas, 69372 Lyon, Cedex 08, France; Radiation Medical Science Center, Fukushima Medical University, 1 Hikarigaoka, Fukushima, 960-1295 Japan; Psychological and Behavioral aspects of Life after Cancer, Danish Cancer Society Research Center, Strandboulevarden 49, 2100 København Ø, Denmark; Department of Radiation Medical Sciences, Atomic Bomb Disease Institute, Nagasaki University, 1-12-4 Sakamoto, Nagasaki, 852-8523 Japan

**Keywords:** Fukushima, Radiation, Thyroid screening, Dosimetry, Mental health

## Abstract

In October 2013, the Radiation Medical Science Center of the Fukushima Medical University and the Section of Environment and Radiation of the International Agency for Research on Cancer held a joint workshop in Fukushima, Japan to discuss opportunities and challenges for long-term studies of the health effects following the March 2011 Fukushima Daiichi Nuclear Power Plant Accident. This report describes four key areas of discussion -- thyroid screening, dosimetry, mental health, and non-radiation risk factors -- and summarizes recommendations resulting from the workshop. Four recommendations given at the workshop were to: 1) build-up a population-based cancer registry for long-term monitoring of the cancer burden in the prefecture; 2) enable future linkage of data from the various independent activities, particularly those related to dose reconstruction and health status ascertainment; 3) establish long-term observational studies with repeated measurements of lifestyle and behavioural factors to disentangle radiation and non-radiation factors; and 4) implement primary prevention strategies targeted for populations affected by natural disasters, including measures to better understand and address health risk concerns in the affected population. The workshop concluded that coordinated data collection between researchers from different institutes and disciplines can both reduce the burden on the population and facilitate efforts to examine the inter-relationships between the many factors at play.

## Background

On October 17 2013, the Radiation Medical Science Center of the Fukushima Medical University and the Section of Environment and Radiation of the International Agency for Research on Cancer held a joint workshop in Fukushima, Japan. The workshop brought together a multidisciplinary team of researchers to share state-of-the-art research related to radiation epidemiology, dosimetry, and mental health and discuss the opportunities and challenges for long-term studies of the health effects following the March 2011 Fukushima Daiichi Nuclear Power Plant Accident. While many topics discussed at the workshop have been raised in earlier publications such as [1-5], the workshop organizers felt it would be useful to share the perspectives communicated during the workshop on four key areas - thyroid screening, dosimetry, mental health, and non-radiation risk factors of chronic disease - related to current, on-going projects in Fukushima [6].

## Thyroid screening

A large-scale thyroid ultrasound screening program targeting approximately 360,000 children (ages ≤18 years) residing in the Fukushima prefecture at the time of the incident began in October 2011 [5,6]. The program is part of the Fukushima Health Management Survey designed to initially establish baseline thyroid status and then monitor participants over their lifetimes. The survey program responds to widespread public concern about thyroid cancer risk following the accident. Based on the Chernobyl accident, radiation-related thyroid cancers would not be expected less than 3–4 years after the accident [7]. The thyroid ultrasound screening program in Fukushima benefits from a design that includes an assessment of baseline thyroid health immediately after the accident, well before any radiation-effect would be expected. Nonetheless, population concerns need to be adequately addressed as the baseline incidence of thyroid cancer is predicted to increase substantially as a function of introducing screening into the population [8]. Further challenges may remain to disentangle a screening effect from that of radiation in the medium- and long-term; particularly if follow-up and adherence to the screening is dependent on the level of radiation exposure received. Workshop participants noted that although the survey establishes important baseline information, there may still be implications of identifying, via screening with improved diagnostic techniques, thyroid diseases that might otherwise never have been detected, in particular an excess of very small or asymptomatic (occult) papillary thyroid cancers for which active surveillance rather than treatment intervention may be the best option [9]. Integrating the thyroid screening program with other components of the Fukushima Health Management Survey [6] (e.g., linkage with the Comprehensive Health Check and Mental Health and Lifestyle Survey) can help to understand the broader long-term physical and mental health of targeted participants undergoing screening.

## Dosimetry

Estimating individual radiation doses among clean-up workers (on-site workers as well as off-site workers) and the general population alike is essential for studies of the long-term health effects following the accident. It is indispensable to link results of the clean-up workers’ dose monitoring to their health check-up data so that it can be used for further follow-up studies. Dose reconstruction efforts among Fukushima residents, such as those described in [2,10] are based on a combination of environmental measurements, whole body counting, personal dosimeter measurements, and self-reported location and dietary patterns (collected as part of the Fukushima Health Management Survey). Based on data from 421,394 Survey participants reported at the 16th Meeting of Health Management in Fukushima Prefecture, estimated external effective dose in the first four months after the accident was <3 mSv for 99% of participants (excluding workers with occupational radiation exposures, including but not restricted to those from Fukushima Daiichi Nuclear Power Plant) in regions other than the most highly contaminated area, where 97% of the non-occupationally exposed population is estimated to have received an effective dose < 3 mSv [11]. Other studies have similarly demonstrated low-dose exposure levels among the residents in Fukushima, including evacuees, through direct and indirect measurement [12-14].

In 2013, UNSCEAR provided preliminary dose estimates for the general population based on environmental measurements and human transfer models [15]. The UNSCEAR report noted the lack of internal measurements at the time general population doses were evaluated; based on subsequently available internal measurements, the authors of the report suspect their estimates may exceed true exposures. Doses were estimated for three age- (infants, 10 years, and 20 years) and location groups (by increasing exposure: “non-evacuated areas”, “precautionary evacuated areas” and “deliberately evacuated areas”). Prefecture-average thyroid dose estimates across these areas range from 0.5-83 mGy with the highest estimates for infants and individuals living in the deliberately evacuated areas. Dose estimates to the red bone marrow and breast range from less than 2–10 mGy across the three areas.

Multiple institutions are involved in dose-reconstruction, possibly acting independently of one another. Workshop participants encouraged the comparison of dose estimates made by different groups for the same individuals (presumably based on somewhat different information and model assumptions). This could be challenging, however, if the various systems rely on different personal identifying information, thus making it difficult to link individuals across studies. Workshop participants also emphasized the importance of calculating organ-specific dose (rather than effective dose) to estimate cancer and non-cancer disease risk as a function of dose in future epidemiologic studies [16]. A detailed set of recommendations for estimating dose following radiation accidents such as Fukushima are described in [17].

## Mental health

Mental health is a core component of the Fukushima Health Management Survey, incorporating both research and clinical (including counselling) components [6]. Meeting participants discussed two broad categories related to mental health, infrastructure and the impact of the accident on the population’s mental health. Infrastructure largely concerns the disruption and subsequent re-establishment of mental health services in disaster-affected areas. Workshop participants highlighted a multitude of factors including trauma, quality of life among the evacuees, stigma and fear/anxiety about radiation exposures. These factors can affect mental health in both the short- and long-terms and can lead to physical symptoms that may be attributed exclusively to the radiation exposure if not properly studied. Participants stressed the importance of conducting long-term studies of psychosocial health following the disaster.

## Changes in non-radiation risk factors

Directly related to mental health is the question of how non-radiation factors, specifically lifestyle and behavioural factors, may alter health of the population in the long-term. There is information that continued fear related to radiation exposure may lead parents and teachers to keep children indoors and that many people continue to avoid locally grown fruits and vegetables, favouring instead food (even if processed) produced elsewhere [18,19]. Many of those evacuated, due to elevated radiation levels or the devastation of the tsunami and earthquake, transitioned from farming communities to urban settings. Longitudinal studies of lifestyle and behavioural factors (e.g., diet, physical activity, smoking and alcohol consumption) that may be related to persistent stress can establish whether there are changes in these factors and examine whether any such changes are associated with long-term risk of outcomes such as cancer and cardiovascular disease. Short-term comparisons of population health before and after the disaster suggest a decline in health status as measured by waist circumference and several metabolic factors [20] but longer-term data based on larger samples are needed.

## Conclusions

The four key areas discussed here - thyroid screening, dosimetry, mental health, and non-radiation risk factors - are closely related. Examples of this inter-relationship include the impact of mental health on long-term changes in other cancer risk factors or the impact of thyroid cancer screening on mental health. Four recommendations given at the workshop were to: 1) build-up a population-based cancer registry for long-term monitoring of the cancer burden in the prefecture; 2) enable future individual linkage of data from the various activities currently conducted independently, in particular integrating the different components of the health survey [6] with internal and external dose estimation efforts outside of the health survey and newly developing disease and mortality registries; 3) establish long-term observational studies with repeated measurements of lifestyle and behavioural factors to disentangle radiation from other factors; and 4) implement primary prevention strategies targeted for populations affected by natural disasters, including measures to better understand and address health risk concerns in the affected population. A resounding conclusion from the workshop was that coordinated data collection between researchers from different institutes and disciplines can both reduce the burden on the population and facilitate efforts to examine the inter-relationships between the many factors at play.

## Abbreviations

mSv: Millisievert
mGy: Milligray
UNSCEAR: United Nations Scientific Committee on the Effects of Atomic Radiation
